# Sustainability of exercise-induced benefits on circulating MicroRNAs and physical fitness in community-dwelling older adults: a randomized controlled trial with follow up

**DOI:** 10.1186/s12877-024-05084-0

**Published:** 2024-05-30

**Authors:** Li-Yuan Huang, Ai Yin Lim, Chih-Chin Hsu, Yun-Fang Tsai, Tieh-Cheng Fu, Yu-Chiao Shyu, Sheng-Chiao Peng, Jong-Shyan Wang

**Affiliations:** 1 Department of Nursing, Keelung Chang Gung Memorial Hospital, Keelung, Taiwan; 2 College of Medicine, Institute of Clinical Science, Chang Gung University, Taoyuan, Taiwan; 3 Healthy Aging Research Center, Graduate Institute of Rehabilitation Science, Chang Gung University, Kwei-Shan, 259 Wen-Hwa 1 Road, Taoyuan, 333 Taiwan; 4 Community Research Center, Keelung Chang Gung Memorial Hospital, Keelung, Taiwan; 5 Department of Physical Medicine and Rehabilitation, Keelung Chang Gung Memorial Hospital, Keelung, Taiwan; 6 School of Nursing, College of Medicine, Chang Gung University, Taoyuan, Taiwan; 7 Research Center for Chinese Herbal Medicine, College of Human Ecology, Chang Gung University of Science and Technology, Taoyuan, Taiwan

**Keywords:** Aerobic exercise, Physical fitness, Body composition, Pathway analysis

## Abstract

**Background:**

Circulating miRNAs (c-miR) have been shown to be potential biomarkers in sarcopenia, but the miRNAs response to aerobic exercise in older people remains inconclusive. We sought to examine the exercise benefits on physical fitness and miRNAs, and to explore the mediating effect of miRNAs on training-induced fitness changes.

**Methods:**

This controlled trial recruited 58 community-dwelling older adults and randomized them into exercise group (EX) and control group (CON). EX received 8-week supervised moderate intensity cycling training 3x/week. C-miR expression (c-miR-21, c-miR-126, c-miR-146a, c-miR-222), physical fitness (body composition, cardiorespiratory fitness, muscular fitness) and physical activity level (PAL, measured as in daily step counts) were evaluated at baseline, post-training, and post-16-week follow-up. The mediating effect of miRNA expression onto exercise-induced physical fitness change was determined by causal mediation analysis (CMA).

**Results:**

Exercise significantly improved body fat and cardiorespiratory fitness in older people while maintaining muscle mass and strength, and augmented expression of c-miR-126, c-miR-146a, and c-miR-222 for up to 16 weeks post-training. Notably, older people in EX had substantially higher daily step counts than CON throughout the study even after the active training period. However, CMA revealed no significant indirect effect but a potential mediating effect of c-miR-21, but not the rest, onto the body composition, cardiorespiratory fitness, and lower limb strength.

**Conclusion:**

An eight-week supervised MICT program promoted a higher level of physical activity up to 16 weeks post-training, which induces better cardiorespiratory fitness and resists decline in muscular measures. C-miRNA, especially c-miR-21, potentially mediates the training effect upon fitness.

**Supplementary Information:**

The online version contains supplementary material available at 10.1186/s12877-024-05084-0.

## Introduction

Age-related deterioration in physical fitness, substantially escalated by insufficient physical activity (PA), has a notorious impact on the older population [1]. Declines in muscular and cardiorespiratory fitness are associated with a greater risk of disability in the older population [2]. Yet, older adults are among the groups with the lowest level of PA [3], which puts them at twice higher mortality risk compared to their more active peers [4]. A recent global report urged policies to be made to increase the population level of PA if were to fulfill the WHO 2025 global PA target, as to reduce the 10% relative probability of insufficient PA [3]. Although a structured exercise program has strong evidence in fighting this aging-induced debilitation [5], how to initiate and sustain the PA level in older adults remains a challenge to public health and the healthcare sector.

Physical exercise is a distinguished measure to battle sarcopenia [6], cardiovascular diseases [7], metabolic syndrome, functional decline, etc. [8]. Maintaining high levels of physical exercise throughout one’s life slows the aging-induced deterioration which is more prominent in inactive individuals [9]. PA guidelines emphasize the necessity to prescribe exercise to both fit and frail older people, and suggest more investigation to discover the optimal level of activity [10]. The older population generally exhibits more comorbidities and lower adherence to exercise programs, making it more challenging to observe exercise-induced modulations at a physiological level. Moreover, a persistent active lifestyle is essential because detraining effect on cardiorespiratory fitness and muscle strength was notably more pronounced in the older population compared to young or middle-aged adults [11, 12].

Understanding the physiological adaptation towards exercise, such as epigenetic regulation, and how it connects with physical performance would facilitate the design of exercise prescription [13].Growing evidence suggested miRNAs as exercise biomarkers and their potential roles as physiological mediators of exercise-induced physical benefits [14]. Circulating miRNAs (c-miRNAs) in plasma or serum exist in exceptionally stable forms, and influence protein translation by regulating gene expression in a wide range of biological processes at the posttranscriptional level [15–17]. Expression profile of myo-related, cardiac-target and pro-inflammatory c-miRNAs could be modified by acute aerobic exercise and prolonged exercise training [18]. However, due to the concerted nature of c-miRNAs, ongoing research is working conscientiously to pinpoint the exclusive c-miRNAs.

Among the numerous c-miRNAs responsive to exercise, c-miR-21, c-miR-126, c-miR-146a, and c-miR-222 have been noted for their specific response to endurance exercise and correlated with skeletal muscle differentiation, generalized angiogenesis activities, systemic anti-inflammation response, and cardiac remodeling, respectively [13, 19]. Elevated levels of c-miR-21 have been observed in aging tissues and were correlated to inflammation [20], but potentially downregulated by regular exercise [21]. c-miR-126 is known for its role in angiogenesis, vascular function, and endothelial cell maintenance and was recently suggested as an exercise indicator for cardiovascular prescriptions [19]. While moderate-intensity exercise is positively associated with vascular function, we would like to investigate the potential correlation between exercise-induced changes in molecular level and aerobic fitness. c-miR-146a is involved in chronic low-grade inflammation, a hallmark of aging. Regular physical activity has been shown to decrease c-miR-146a levels in circulating cells, indicating a potential anti-inflammatory effect. C-miR-222 is known to regulate cell proliferation, apoptosis, and angiogenesis. Exercise modulates the expression of c-miR-21, c-miR-126, c-miR-146a, and c-miR-222 in older adults, contributing to various physiological responses that promote healthy aging and reduce the risk of sedentary behavior. Our study aimed to elucidate the specific mechanisms through which exercise modulates these miRNAs and their implications for aging and health.

To contribute to the above-mentioned research gaps, we first aim to explore the immediate post-training and prolonged follow-up effect of aerobic exercise in altering older people’s physical fitness and their PA level; then to explore the association of exercise-induced changes in the expression of c-miRNA and physical fitness progress.

## Methods

### Study design

This is an assessor-blinded, parallel, randomized controlled trial conducted in a tertiary care hospital and involved community-dwelling older adults aged 60 and above. This study is conformed to the CONSORT 2010 guidelines [22]. The study was approved by the ethics committees of the Chang Gung Memorial Hospital (IRB number 201800289A3; clinical trial registration number NCT04940884, 28,062,021) and all subjects provided written informed consent before participation. Participants were randomized to exercise group (EX) or control group (CON) and assessed at baseline (T0, before the randomization), then reassessed at week 8 (T1, upon completion of the exercise training) and week 24 (T2, endpoint of the study). Outcomes included the change of (i) physical fitness, (ii) c-miRNA levels and inflammatory markers, (iii) physical activity level, and (iv) health-related quality of life.

### Participants

We recruited older adults from February 2021 to December 2021 at Chang Gung Memorial Hospital, Keelung Branch, Taiwan. Eligibility criteria were older adults aged at least 60 years old, with no habit of regular physical activity, and had a walking speed of > 0.8 m/sec as tested on 3-m shuttle walk test. Exclusion criteria were a Mini-Mental State Examination (MMSE) score of < 24, diagnosed with spine/lower extremity degenerative joint disorder, atrial fibrillation/flutter, second/third degree heart block, history of life-threatening ventricular arrhythmias, recent (< 4 weeks) unstable angina, recent (< 4 weeks) myocardial infarction or coronary revascularization, uncontrolled diabetes mellitus, anemia (hemoglobin concentration ≤ 12 g/dL in men and ≤ 11 g/dL in women), severe chronic obstructive pulmonary disease, or symptomatic cerebrovascular disease within 12 months, collagen vascular disease, alcohol or drug abuse during the previous 12 months, estimated glomerular filtration rate of < 30 mL/min/1.73 m^2^, acute hepatitis, malignancies, or sarcopenia as according to Asian Working Group for Sarcopenia [6], or presented with conditions in line with the ACSM’s guideline for contraindications to exercise [5].

### Intervention

Eligible older adults were randomly allocated to the EX or CON at a ratio of 1:1, based on a computer-generated and concealed allocation schedule. All participants attended one session of health talk and were advised to achieve at least 8000 steps every day during the trial period. Daily step counts were recorded via a wearable activity tracker (smartwatch), along with weekly reminder calls for the first eight weeks. The EX received additional 24 sessions of supervised cycling training for the first eight weeks. The cycling exercise was performed three times per week on an Ergoselect 150P bicycle ergometer (ergoline GmbH, Germany) at 70% of maximum predicted heart rate for 30 min per session under supervision of a physical therapist. Exercise session would be terminated if the participant exhibited signs/symptoms during exercise according to the ACSM guideline [5].

### Physical fitness parameters

#### Body composition

Body composition including fat free mass, skeletal muscle mass and body fat mass were estimated using Inbody 720, multiple frequency bioimpedance analysis (Inbody, South Korea) at T0, T1 and T2. The SKM and BFM were normalized by FFM and body weight (nSKM and nBFM), respectively.

#### Cardiorespiratory and muscular fitness

Participants’ cardiorespiratory fitness (CRF) was tested by the 2-min step test (2MST), while upper and lower limbs strength were assessed using hand grip strength (HGS) and 5 times sit to stand test (5XSST), respectively. 2MST and 5XSST were tested using eFitHealth, a movement analysis system (uCare Medical Electronics, Taiwan), to evaluate lower extremity muscle strength and CRF, respectively. The number of steps derived from the 2MST was utilized for calculating and then presented as estimated metabolic equivalents (METs) to explain daily aerobic capacity [23, 24]. HGS was determined as the mean value from two trials for the maximal grip strength of the dominant hand using an Isoforce GT310 handheld dynamometer (OG Giken, Japan). Bilateral calf circumference (calf_circ) was measured as a clinical indicator for muscle mass.

### Analysis of c-miRNA and inflammatory markers

#### Plasma sampling and RNA extraction

Participants were requested to fast for at least eight hours and avoid strenuous physical exercise for at least 48 h before blood sampling at T0, T1 and T2. Ten mL of venous blood were drawn from cubital fossa venipuncture into tubes containing 3.2% sodium citrate and then was centrifuged to obtain platelet-poor-plasma (PPP). The PPP samples were processed and stored at -80 °C, until RNA extraction.

The total RNA was extracted from plasma samples using a Direct-zol™ RNA MiniPrep Kit (Zymo Research, USA) according to the manufacturer protocol. The selected four candidate miRNAs c-miR-21, c-miR-126, c-miR-146a, and c-miR-222 were analyzed. miR-39 of Caenorhabditis elegans (Cel-miR-39) was added to all samples, as an exogenous control to the subsequent qPCR procedure. Detailed procedure is attached as Supp. Material 1.

#### Selection of candidate miRNAs

##### Reverse transcription and c-miRNA quantification

This study evaluated the level of plasma c-miR-21, c-miR-126, c-miR-146a and c-miR-222 on a T100™ Thermal Cycler (Bio-Rad, USA). Sample miRNAs were transcribed into cDNA via miRNA specific reverse transcription reaction. The miRNA expression analysis was performed using miRCURY LNA SYBR Green PCR kit (Qiagen GmbH, Germany) following the manufacturer’s instructions. Participants’ plasma c-miRNA expression levels were normalized by the Cel-miR-39 level (detailed protocol in Supp. Material 1).

##### Inflammation-related cytokine activities

Five mL of blood was obtained and centrifuged into PPP which then stored in 500 μl aliquots at -80 °C, until ELISA assay. Circulating cytokines (IL-1α, IL-1β, IL-6, IL-10, and TNFα) in the plasma were quantified using Multiplex Human Cytokine Panel 1 assay kit (Boster Biological Technology, USA).

### Physical activity level

All participants were issued with a wearable activity tracker (Smartwatch WDI08, WisDat Inc., Taichung, Taiwan) and were instructed to wear throughout the day, except occasions that would expose the watch to significant amount of water. Weekly calls were made in the first eight weeks, to remind all the participants to accomplish the goal of 8000 steps/day. Data was presented as an average of daily steps for seven days.

### Quality of life

Health related-quality of life (HR-QoL) was evaluated at T0 and T2 using the Traditional Chinese version of the SF-36, a reliable and valid tool [25]. The physical component subscale (PCS) and mental component subscale (MCS) scores were used as outcomes.

### Statistical analysis

Descriptive statistics and univariate/multivariate analysis were executed using SPSS 25.0. We tested the normality of continuous dependent variables by performing Kolmogorov–Smirnov test. The baseline values between two groups were compared using Independent Student’s t-test and Chi-square test for continuous and categorical variables, respectively. To examine the training effect from T0 (baseline) to T1 (immediate) and T2 (follow up), the within- and between-group difference were tested by mixed ANOVA, with Bonferroni post hoc analysis if applicable, and were adjusted for the age, sex, BMI and comorbidities. Data that did not normally distributed were analyzed by corresponding non-parametric tests.

We further explored the mediating role of c-miRNA (at T1) in prolonged training-induced fitness benefits (at T2) via causal mediation analysis using R software (version 4.0.3) lavaan package. Potential mediational pathways (Supp. Figure 1) were identified by the following procedures with regression analyses according to Baron and Kenny [26]. Sobel’s test was used to examine the significance of the indirect effect [27]. Indirect effects were measured by the product of two path coefficients (m_1_: intervention-mediator and m_2_: mediator-outcome). The standard errors of indirect effects were estimated by bootstrapping method, which no assumption about normal distribution of path coefficients was needed. The amount of mediation was presented by the proportion of total effect (i.e., both direct and indirect effects) explained by indirect effect. All statistical analysis were two-tailed and considered significant at *p* < 0.05.

## Results

**Fig. 1:**
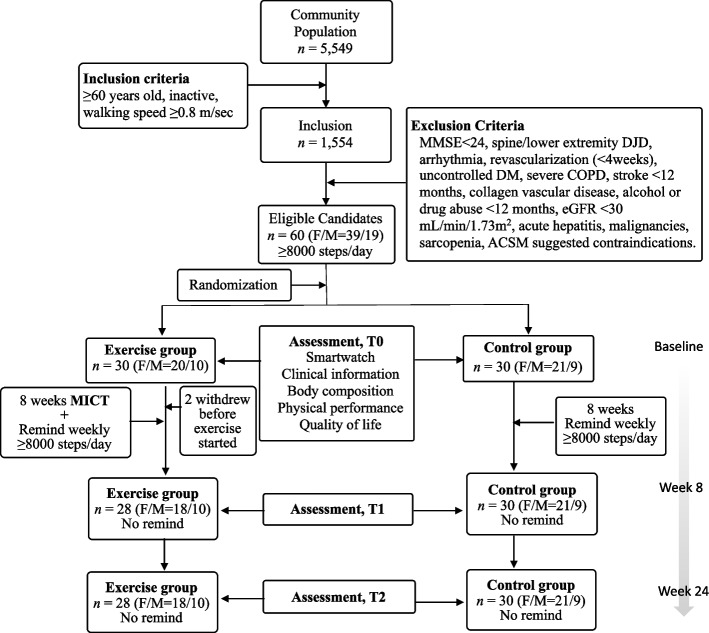
Flow Diagram of Selected Participants. ACSM, American College of Sports Medicine; COPD, chronic obstructive pulmonary disease; DJD, degenerative joint disorder; DM, diabetes mellitus; eGFR, estimated glomerular filtration rate; F/M, female/male; MI, myocardial infarction; MICT. moderate-intensity continuous training; MMSE, mini-mental status examination; stp/d, step per day; WS, walking speed

Figure 1 shows the study flow from initial recruitment to final analysis. A total of 5,549 community residents in the Cohort study were screened, in which 1,554 people met the inclusion criteria but 1,494 people were subsequently excluded, resulted in 60 people being eligible for the study. The participants were randomly assigned to either EX or CON. Two participants in the EX withdrew before the exercise program started. Hence, the final number of participants was 28 in the EX and 30 in the CON.

Table 1 shows the baseline characteristics of all participants and the physical fitness parameters at three timepoints, respectively. Both groups have no significant difference in mean age, gender distribution, BMI, comorbidities, current medication, and physical fitness.

**Table 1:** Baseline characteristics of participants

	Exercise group (*n* = 28)	Control group (*n* = 30)	p
Age, years	67.1 ± 4.19	66.1 ± 5.87	0.405
Female	18 (64.3)	21 (70.0)	0.643
BMI, kg/m^2^	24.3 ± 3.78	24.5 ± 9.08	0.813
Comorbidities			
Hypertension	9 (32.1)	8 (26.7)	0.647
Type 2 DM	4 (14.3)	3 (10.0)	0.701^a^
Hyperlipidemia	10 (35.7)	8 (26.7)	0.457
Medication			
ACEI/ARB	6 (21.4)	4 (13.3)	0.499^a^
β-Blocker	3 (10.7)	0 (0)	0.106^a^
CCB	4 (14.3)	3 (10.0)	0.701^a^
Diuretics	3 (10.7)	1 (3.3)	0.345^a^
OHA	4 (14.3)	3 (10.0)	0.701^a^
LLA	10 (35.7)	7 (23.3)	0.301
Body composition			
SKM, %	37.49 ± 4.34	36.58 ± 4.36	0.433
BFM, %	33.33 ± 8.28	31.74 ± 7.37	0.442
Cardiorespiratory fitness			
2MST, steps	95.46 ± 16.45	99.87 ± 15.93	0.305
Muscular fitness			
HGS, kg	28.66 ± 12.03	29.20 ± 9.87	0.854
5XSST, s	10.75 ± 3.01	11.68 ± 2.72	0.224

*Abbreviations*: *BMI* body mass index, *DM* diabetes mellitus, *ACEI* angiotensin-converting enzyme inhibitor, *ARB* angiotensin receptor blocker, *CCB* calcium channel blocker, *OHA* oral hypoglycemic agent, *LLA* lipid-lowering agent

Data are presented as mean ± standard deviation or n (%)

^a^Results are based on Fisher’s Exact Test

### Training effect on physical fitness

**Fig. 2:**
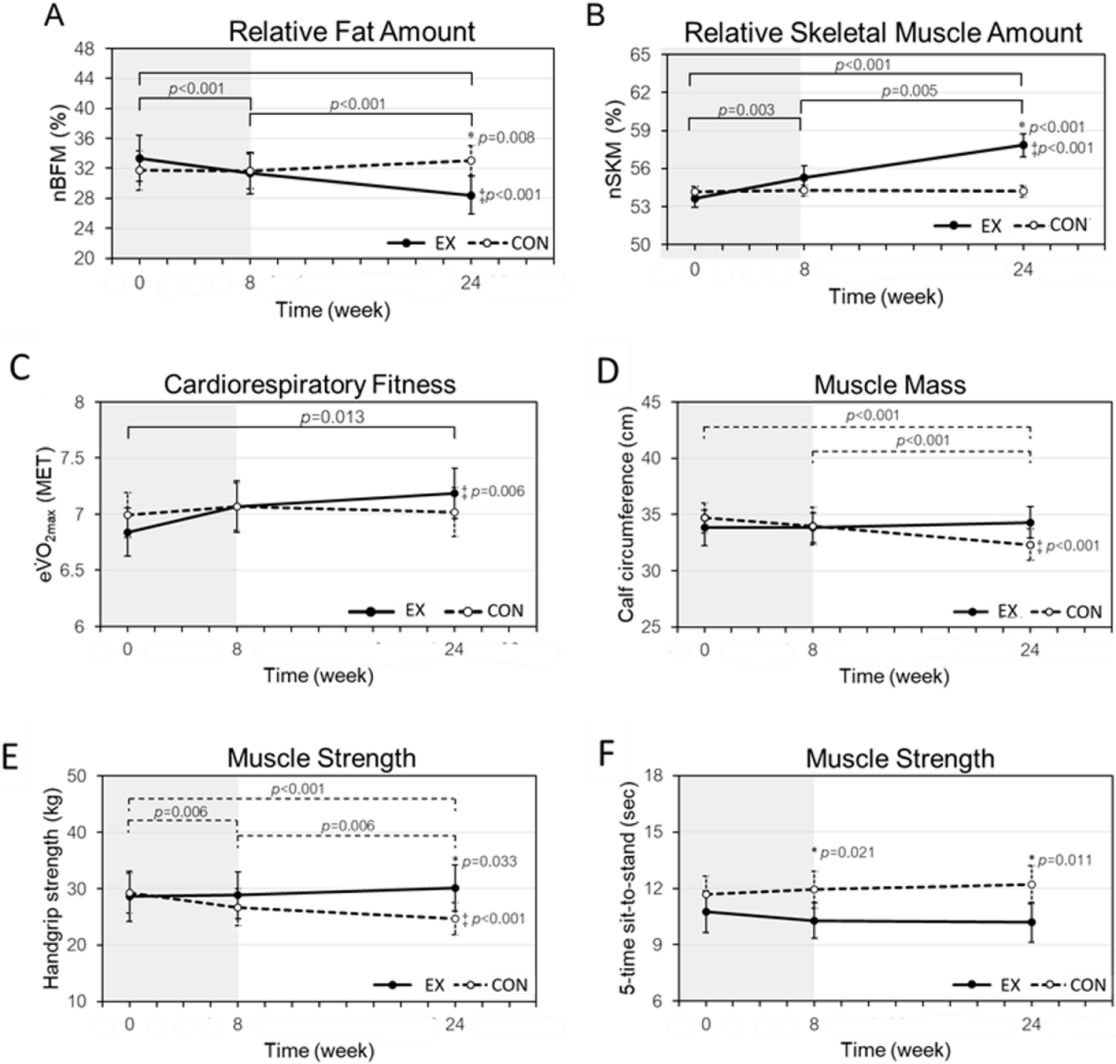
**A** normalized body fat mass (nBFM) representing relative fat amount in SET participants significantly decreased during follow up and was significantly less than HET participants at the end of follow up. **B** normalized skeletal muscle mass (nSKM) representing relative skeletal muscle amount increased significantly after MICT, and EX participants had significantly greater nSKM than those in CON participant. **C** estimated metabolic equivalent (MET) significantly increased during follow up in EX participants. **D** calf circumference in CON participants representing muscle mass significantly decreased during follow up, and significantly less muscle mass than EX participants. **E** handgrip strength in CON participant significantly decreased during, and significantly less muscle mass than EX participants. **F** EX took significantly less time than CON participants to complete 5-time sit-to-stand test. *: significant group effect; ‡: significant time x group interaction by mixed ANOVA and Bonferroni post-hoc test to assess differences between each of the assessment time point

There was significant time*group interaction in nBFM but no significant interaction seen for the other physical fitness parameters. For nBFM, simple effects for group (EX vs CON) were observed at T2 (*p* = 0.008). Simple effects for time were significant in EX only (*p* < 0.001), showing that participants improved in body composition after eight weeks of cycling exercise (T1) and at 24th week post training (T2) (Fig. 2A, B). For nSKM, main group effect was seen at T2 (*p* < 0.001) and main time effects were seen in EX between T0 ~ T1, T1 ~ T2, T0 ~ T2 (*p* < 0.01 for all). For CRF presented in METs, significant main effects for group were observed at T2 (*p* = 0.006). Significant main time effects were seen in EX between T0 and T2 (*p* = 0.013) (Fig. 2C). The participants’ calf-cir and HGS had no significant group effect at any timepoints. EX did not show significant time effect, whereas CON showed significant reduction in calf-cir and HGS from T0 to T2, and from T1 to T2 (both *p* < 0.001) (Fig. 2D, E). Lower limb strength, as measured in 5XSST, of both groups had no significant time effects but showed significant group effect at T1 (*p* = 0.021) and T2 (*p* = 0.011) (Fig. 2F).

### Plasma levels of candidate miRNAs

**Fig. 3:**
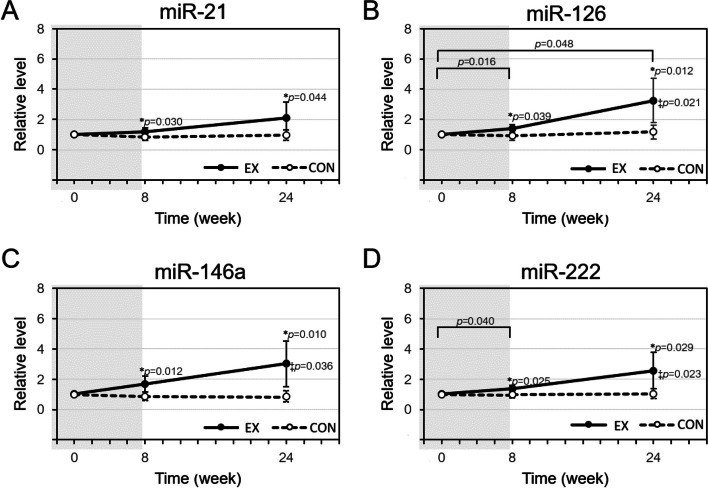
**A** a non-significant increase of cmiR-21 was observed in EX participants and was greater than those in CON participants during follow up. **B** a significant increase of cmiR-126 in EX participants and was significantly greater than those in CON participants during follow up. **C** a significant increase of cmiR-146a was observed in EX participants and was greater than those in CON participants during follow up. **D** a significant increase of cmiR-222 in EX participants and was significantly greater than those in CON participants during follow up. *: significant group effect; ‡: significant time x group interaction by mixed ANOVA and Bonferroni post-hoc test to assess differences between each of the assessment time point

At baseline, the levels of all selected miRNAs were all detectable in plasma and presented relatively low expression. There was no significant difference between both group at baseline and no group*time interaction was seen. Main effects for group were observed at T1 and T2 for all c-miRNA (*p* < 0.05), where EX had higher fold change than CON. After eight weeks of cycling exercise and 16 weeks of follow up, circulating plasma levels of miR-126 (2.24 mean fold change, *p* = 0.021), miR-146a (2.01 mean fold change, *p* = 0.036), and miR-222 (1.55 mean fold change, *p* = 0.023) were significantly up-regulated from T0 to T2 (Fig. 3).

### Inflammatory-related cytokines activities

Circulating IL-1α, IL-1β, IL-6, IL-10, and TNFα in both groups showed neither within- or between-group significant difference at the three time points of assessment (Supp. Figure 2).

### The changes of physical activity level

**Fig. 4:**
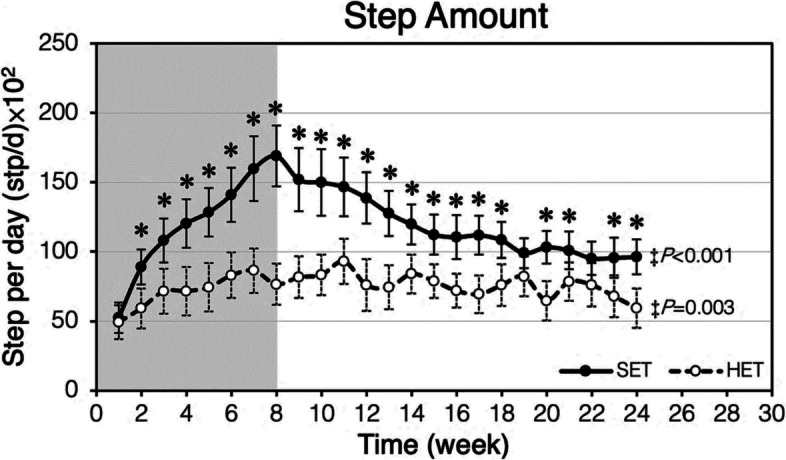
Mean walking steps per day (stp/d) in a week representing the step amount in the week during follow-up

By tracking the daily step counts, the participants in both groups had similar physical activity at the beginning of the study (averagely 5613 steps and 5019 steps at week 1, EX and CON, respectively). The daily step counts of EX steeply increased for the following eight weeks in accordance with the cycling exercise program, subsequently declined over the next follow up period. EX had significantly higher step counts at 24th week than 1st week (*p* < 0.001), whereas CON showed no difference (Fig. 4).

### Quality of life

Although the self-administered SF-36 did not show significant within- and between-group difference at the beginning and the end of the study, there is a trend of improving physical and mental component score in both groups (Supplemental Fig. 1).

### Causal mediation analysis

**Fig. 5:**
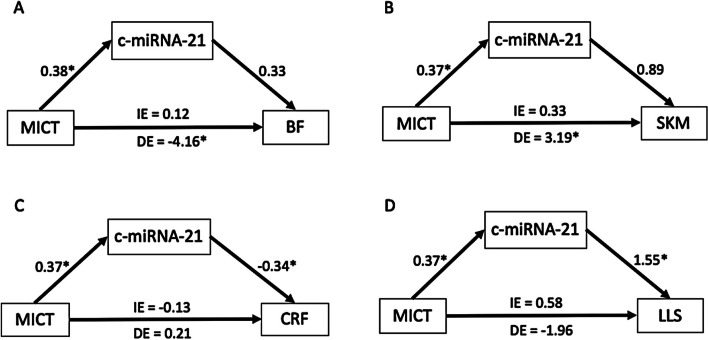
Pathway Analysis for association between MICT and physical fitness. **A** significant direct effect of MICT to normalized body fat mass adjusted by c-miRNA-21. **B** significant direct effect of MICT to normalized skeletal muscle mass adjusted by c-miRNA-21. **C** significant m1 and m2 path, suggesting association between c-miRNA-21 and training/CRF. **D** significant m1 and m2 path, suggesting association between c-miRNA-21 and training/lower limbs strength. *: significant association

In the CMA model, the effect of training was significant on body composition adjusting for all four candidate c-miRNAs. MICT was significantly associated with nSKM (DE = 3.19, 95%CI = 1.76 ~ 4.62) and nBFM (DE = -4.16, 95%CI = -7.98 ~ -0.34) adjusted for c-miR-21 (Fig. 5). Similar result was observed in the pathways mediated via c-miR-126, c-miR-146a, and c-miR-222 (Supp. Table 1). Mediation analysis revealed insignificant indirect effect (IE) even though the association between MICT and miRNAs (m1) were all significant. Particularly, the pathway of MICT on CRF and 5XSST mediated via c-miR-21 did not show significant direct/ indirect effect, but both m1 and m2 were significant (m1 = 0.37, m2 = -0.34 for CRF, Fig. 5B; m1 = 0.37, m2 = 1.55 for 5XSST, Fig. 5).

## Discussion

Main findings showed training effect on the change of body composition (increased skeletal muscle mass with reduced body fat) and at molecular level (c-miR-126 and c-miR-222 expression level increase after 8 weeks which sustained till week 16 post training). The association between the exercise-induced changes in physical parameters and c-miRNAs, as evaluated by mediation analysis, revealed significant associated between c-miR-21 and body composition.

The older adults in this study were at the > 75th percentile value for their age and sex in performing the 2MST, with an estimated METs of 6 ~ 7. Although the improvement after training was statistically significant, the total METs increment was less than one MET, which may not impose on clinical significance [28]. Older people in this study maintained their body weight but those that underwent MICT not only increased in skeletal muscle mass but also substantially reduced in body fat at the end of the study. Whereas their counterparts in control group had no change in body compositions. Interestingly, the assessments of muscle mass (calf-cir) and muscle strength (HGS) were maintained in the EX but declined in the CON throughout the trial. Older adults with lower level of physical activity demonstrated a reduction of HGS, as much as 4.34 kg, after 24 weeks even though the skeletal muscle mass was maintained. Although yet to meet the criteria of dynapenia, the HGS at 24th weeks are at lower percentile according to the age and gender [29]. Aging community-dwellers without supervised MICT had low exercise adherence and developed a trend towards dynapenia. Nevertheless, the declination of hand grip strength was potentially influenced by the presence of diseases such as diabetes, hypertension, etc. and the consumption of medication [30]. A cross sectional study reported that long-term aerobic exercise attenuates age-related deterioration in muscle strength in older adults [31]. Our results of short term supervised MICT on previously sedentary older adults were in line with their finding and appears to be practical in maintaining muscular performance which sustained for up to 16 weeks post training. In this study there was no significant detraining effect in the physical fitness in the EX group attributed to the maintained higher level of physical activity. Nevertheless, our physical assessment was a field test which could be less sensitive in picking up trivial changes as compared to laboratory testing.

This study demonstrated the efficacy of an eight-week supervised exercise program in promoting active lifestyle where daily step counts were traced up to 16 weeks post training. Verbal encouragement was given weekly to all the older adults at the initial eight weeks, yet only those received MICT three times per week engaged higher level of physical activity throughout the study. Although behavioral adaptation was not measured in this study, the finding may suggest an exercise psychology involvement. Exercise psychologists introduced Affective-Reflective Theory of physical inactivity and exercise in recent years, emphasizing the behavioral interventions (other than the conversation-based) should include physical activity delivered by well-trained exercise specialist in tailored exercise load control [32]. The cycling exercise program was carried out in the rehabilitation department in a hospital, supervised by a physical therapist and overseen by the rehab physician. We believed that the pro-exercise ambience facilitated the transition of being inactive to initiating action in the older people. We evaluated how MICT training modulate the expression of c-miR-146a, c-miR-126 and c-miR-222, which were reported to be correlated with anti-inflammatory effect, generalized angiogenesis activities and cardiac remodeling. These miRNAs increased about 1 to twofold-change in expression 16 weeks post training. C-miR-146a negatively regulates the inflammatory response by targeting TNF receptor-associated factor 6 (TRAF6) and IL-1R-associated kinase (IRAK-1) to inactivate NF-κB in the cytoplasm [33]. Furthermore, miR-146a regulates mitochondria of NOX4 protein expression, thus modulating cellular senescence and redox status which was modifiable through exercise [34]. C-miR-126 and c-miR-222 are both associated with vascular health and endothelial cell integrity. C-miR-126 augments the proangiogenic effects of VEGF and FGF, facilitating the formation of blood vessels by inhibiting the expression of Spred-1, an intracellular inhibitor of angiogenic signaling [35]. Similarly, c-miR-222 affects vascular permeability by regulating adherens junction disassembly, cell migration, and cell morphology, as well as signal transducer and activator of transcription 5A expression [36]. Both c-miRNAs were also reported to be involved in the indirect control of Endothelial Nitric Oxide Synthase (eNOS) which is a key enzyme in producing nitric oxide in the endothelial cells. Previous study reported that miR-222 improved close to twofold change after 8 weeks of training in young athletes [18].

Our investigation on the mediating effect of miRNAs in the causal relationship of training and fitness returned inconclusive finding where the myo-related miRNA, c-miR-21, had effect on the body composition and lower limb muscle performance, but not the other three c-miRNAs. The finding suggests an association of c-miR-21 and body composition, which is in line with previous research [37]. Our previous study suggested that c-miR-146a (inflammation-related miRNA) is associated with age-related sarcopenia [38] Current finding showed increment of c-miR-146a expression after training and pathway analysis suggest a trend of association with body composition. This study excluded participants with a walking speed below 0.8 m/s, thus we could not conclude the training effect in individuals with sarcopenia or dynapenia. Although all candidate miRNAs increased in expression post training and at follow up, only c-miR-21 showed the partially mediating roles on the association between MICT and physical fitness. c-miR-21 is more likely to fine-tune rather than switch on/off the cardiac remodeling [39] and skeletal muscle differentiation [40] in responding to the supervised MICT.

Previous studies suggested that miRNAs regulate cytokine expression [41]. Our findings did not observe a significant difference in all measured cytokines, even though there was a significant post-intervention increment of c-miR222. Our previous study[38] proposed that c-miRNAs are superior to inflammatory cytokines in plasma for serving as critical biomarkers of age-related sarcopenia. Therefore, it could be explained that 8-week exercise and improved level of physical activity for more than four months may not be enough to modulate the miRNA-cytokine regulation. Physical activity may be shown in the fold change of circulating miRNA but not at the post-transcriptional level. Further evidence is needed to explain the potential mediating effect of the epigenetic markers in the exercise-induced physiological adaptations. In this study, community-dwelling adults exhibited PCS and MCS scores in the SF-36 that were relatively lower than the population average [42]. However, over the six-month follow-up period, no significant changes were observed, possibly due to the smaller sample size and high inter-individual variance. Interestingly, the exercise (EX) group showed an improving trend in their mental component but not the physical component, as assessed by the SF-36, despite improvements in physical fitness.

### Study limitation

Several limitations in this study warrant attention. Firstly, the CRF was assessed by using 2MST, which although convenient, could not estimates $\dot{\text{V}}$ O_2max_. We converted the result of 2MST to METs, a more direct measure to explain daily function capability. Secondly, the participants presented with a variety of comorbidities. We recommend refining the eligibility criteria to minimize the impact of confounding factors like comorbidities, which may result in significant variability. In future studies, increasing the sample size and implementing randomization could effectively mitigate the influence of confounding factors. Thirdly, this study did not collect the participants’ dietary information but advised them to maintain their usual diet. Future studies should gather more relevant information such as dietary information, types of physical activity, exercise volumes and other psychosocial measures. Furthermore, a greater number of participants would enhance the investigation of mediation effect of epigenetic markers. Furthermore, a wider spectrum of microRNA expressions is needed to explore the relationship between biological phenomena and physical performances since miRNAs often work in concert. Future study may consider broader involvement of exercise-responsive miRNAs to better elucidate their role as physiological mediators in physical adaptation.

## Conclusions

An eight-week supervised MICT program promoted a higher level of physical activity up to 16 weeks post-training, which induces better cardiorespiratory fitness and resists decline in muscular measures. Circulating miRNA, especially c-miR-21, potentially mediate the training effect upon body composition and CRF but the CMA finding was inconclusive.

### Supplementary Information

Supplementary Material 1.

Supplementary Material 2.

Supplementary Material 3.

Supplementary Material 4.

## Data Availability

The datasets of this study are available from the corresponding author on reasonable request.

## Abbreviations

ACEI: Angiotensin-converting enzyme inhibitor
ACSM: American College of Sports Medicine
ARB: Angiotensin receptor blocker
BMI: Body mass index
calf_circ: Calf circumference
CCB: Calcium channel blocker
CMA: Causal mediation analysis
c-miRNA: Circulating microRNA
COPD: Chronic obstructive pulmonary disease
DJD: Degenerative joint disorder
DM: Diabetes mellitus
eGFR: Estimated glomerular filtration rate
eV ˙O2max: Estimated maximal oxygen consumption
F/M, female/male; HGS: Hand grip strength
LLA: Lipid-lowering agent
MCS: Mental component score
MET: Metabolic equivalent
MI: Myocardial infarction
EX: Exercise group
CON: Control group
MICT: Moderate-intensity continuous exercise training
MMSE: Mini-mental status examination
nBFM: Normalized body fat mass
nSKM: Normalized skeletal mass
OHA: Oral hypoglycemic agent
PA: Physical activity
PCS: Physical component score
QoL: Quality of life
