# Supplementary material for: Sustainability of exercise-induced benefits on circulating MicroRNAs and physical fitness in community-dwelling older adults: a randomized controlled trial with follow up
Source: BMC Geriatr. 2024 May 30;24:473. doi: 10.1186/s12877-024-05084-0 (PMC11137894; doi:10.1186/s12877-024-05084-0)
Supplement: Supplementary file 1 — Supplementary Material 1. [file 12877_2024_5084_MOESM1_ESM.pdf]

Supplementary material 1

RNA Extraction

Whole blood of 10 ml from each subject was sampled at the recruitment, and week 8 as well as week 24 after the recruitment. Specimens were placed in tubes containing 3.2% sodium citrate and were then centrifuged at 1350 rpm for 15 min. The supernatant was further centrifuged at 5000 rpm for 20 min at room temperature to keep plasma platelet count less than 2.5×10^8/ml. The platelet-poor-plasma of 400 μL was placed in a 2 mL safe-lock tube (Eppendorf corp. Hamburg, Germany) containing a mixture of 1200 μL TRIzol (ThermoFisher Scientific Inc., Waltham, MA, USA), 5 μL miR-39 (5×10^{-15} mol/μL) of Caenorhabditis elegans (C-miR-39) as exogenous control, and 2 μg (10 μg/mL in plasma) yeast RNA (Invitrogen, Carlsbad, CA, USA) for 15 min at room temperature. Another 320 μL chloroform was added and placed at room temperature for 5 min. The processed specimen was centrifuged at 11000 rpm for 15 min at 4 °C and 300 μL colorless fluid layer was aspirated to mix with 900 μL iced 100% ethanol overnight at -80 ℃. The prepared specimen was placed into the Direct-zol column (Direct-zol RNA Miniprep, Zymo Research corp., Irvine, CA, USA) and was then centrifuged at 11000 rpm for 30 sec. The column was then transferred to a new collection tube, which was centrifuged at 11000 rpm for 30 sec after mixing with 400 μL RNA wash buffer. DNase I reaction solution of 80 μL (DNase I enzyme 5 μL+DNA digestion buffer 75 μL) was added to the collection tube after discarding the RNA wash buffer and was incubated at room temperature for 15 min. Additional 400 μL of pre-wash buffer was introduced into the tube and was centrifuged at 11000 rpm for 30 sec. The column was then transferred into a new 1.5 mL RNase-free tube and was treated with 80 μL nuclease-free water at room temperature for 2 min. The prepared sample was centrifuged at 11000 rpm for 2 min to elute RNA solution.

Quantification of circulating microRNA levels

Circulating human microRNA-21 (c-miR-21), miR-126 (c-miR-126), miR-146a (c-miR-146a) and miR-222 (c-miR-222) levels at the three different sampling time were analyzed after extraction of RNA. One-step real-time quantitative polymerase chain reaction (RT-qPCR) was performed using a RT-qPCR system (T100^{TM} Thermal Cycler, Bio-Rad Laboratories Inc., Berkeley, CA, USA) to assess plasma miRNA levels.

miRCURY LNA SYBR Green PCR kit (Qiagen GmbH, Hilden, Germany) was used to determine miR-39 from Caenorhabditis elegans (Ce-miR-39), c-miR-21 and c-miR-222. A mixture of 4 μL 5X miRCURY RT SYBR Green Reaction Buffer, 2 μL 10X miRCURY RT Wnzyme Mix and 14 μL of 100 ng total RNA extraction in nuclease-free water were incubated in a 48-well plate at 42°C for 60 min and followed by 95°C to generate cDNA. A mixture containing the above miRNA primers purchased from Qiagen, 10 μL miRCURY SYBR Green Master Mix, 2 μL nuclease-free water and 6 μL 5X generated cDNA template was incubated at 95°C for 2 min, followed by 40 cycles of 95°C for 10 sec and 56°C for 60 sec. The c-miR-21 and c-miR-222 expressions were also normalized by the Ce-miR-39 level.

For determination of Ce-miR-39, c-miR-126 and c-miR-146a expressions, a mixture of 100 ng total RNA extraction, 10 μL TaqMan master mix (ThermoFisher), 5.8 μL nuclease-free water, 0.2 μL universal probe library 21 (10 μM), 0.1 μL RNase inhibitor, self-constructed 1 μL forward primers and 1 μL reverse primers (see the table below), and 2 μL cDNA template were created on ice. All reactions were incubated in a 48-well plate at 95 °C for 3 min, followed by 40 cycles of 95°C for 5 sec, 60°C for 10 sec and 72°C for 1 sec. The c-miR-126 and c-miR-146a expression levels were then normalized by the Ce-miR-39 level.

Determination of inflammatory ctivities

300 μL of serum was diluted by 1:2 ratios. Loaded 50 μL of prepared samples per well and

calibrators in duplicate onto the assay plate (Multiplex Human Cytokine Panel 1, Boster Biological Technology, Pleasanton, CA, USA). Each well containing antibodies captured IL-1α, IL-1β, IL-6, IL-10, and TNFα. A mixture containing biotinylated analyte specific antibodies were added after washing away unbound proteins. The biotinylated antibodies completed the sandwich for each specific arrayed analyte. After washing away unbound biotinylated antibodies, streptavidin horseradish peroxidase was introduced to proportionally reflect the amount of the above captured cytokines. The amount of conjugated enzyme on each location of the array was measured with the addition of a chemiluminescent substrate.
