# Supplementary material for: Sustainability of exercise-induced benefits on circulating MicroRNAs and physical fitness in community-dwelling older adults: a randomized controlled trial with follow up
Source: BMC Geriatr. 2024 May 30;24:473. doi: 10.1186/s12877-024-05084-0 (PMC11137894; doi:10.1186/s12877-024-05084-0)
Supplement: Supplementary file 2 — Supplementary Material 2. [file 12877_2024_5084_MOESM2_ESM.pdf]

Supp. Fig. 1: Conceptual model of causal mediation analysis.

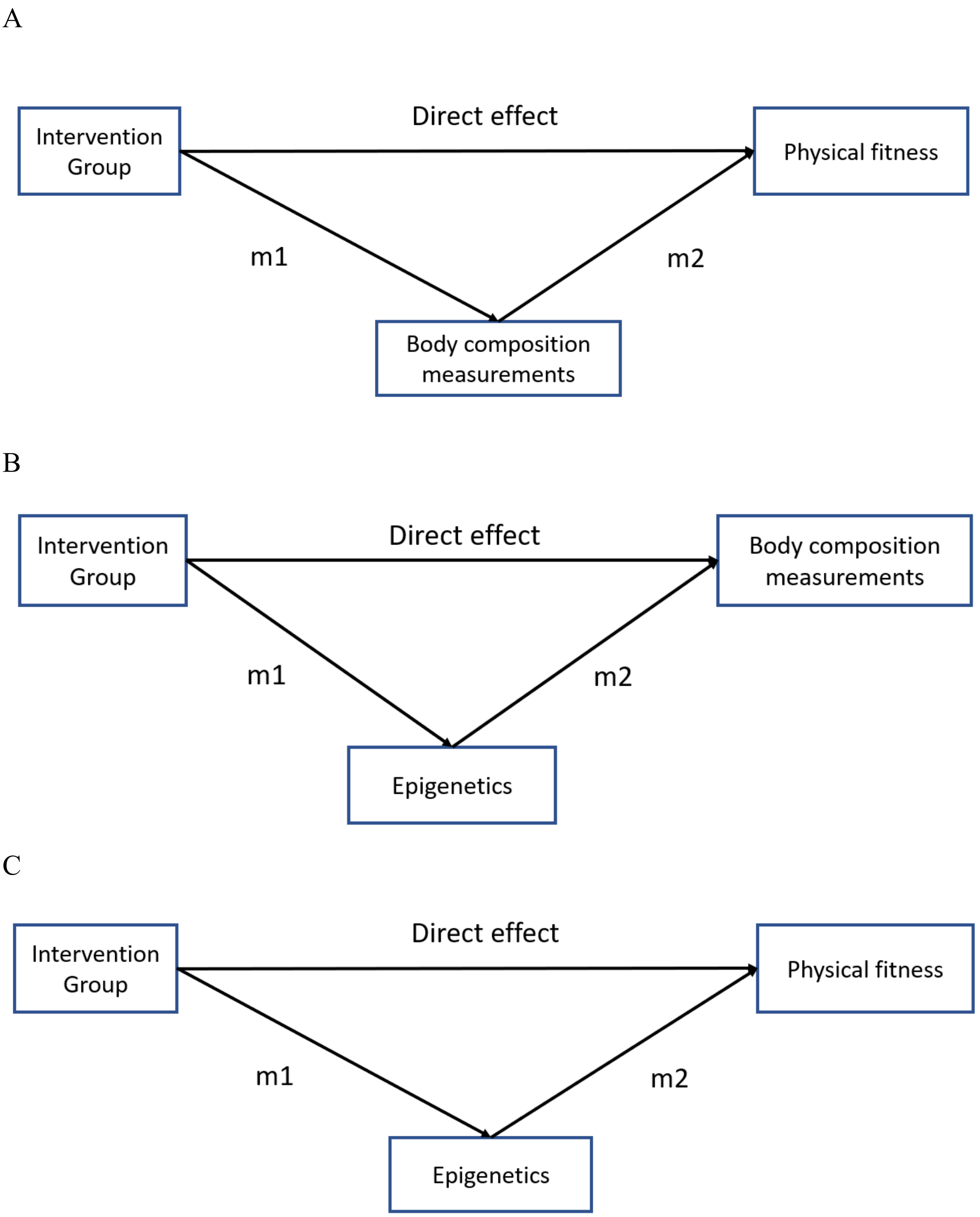

A. Exercise training (Intervention) effects on cardiorespiratory and muscular fitness (physical fitness) and mediating roles of body composition measurements. B. Exercise training (Intervention) effects on body composition measurements and mediating roles of microRNA (epigenetics). C. Exercise training (Intervention) effects on cardiorespiratory and muscular fitness (physical fitness) and mediating roles of microRNA (epigenetics). m_1: pathway coefficient between intervention and mediator; m_2: pathway coefficient between mediator and clinical outcome.
