# Supplementary material for: Sustainability of exercise-induced benefits on circulating MicroRNAs and physical fitness in community-dwelling older adults: a randomized controlled trial with follow up
Source: BMC Geriatr. 2024 May 30;24:473. doi: 10.1186/s12877-024-05084-0 (PMC11137894; doi:10.1186/s12877-024-05084-0)
Supplement: Supplementary file 3 — Supplementary Material 3. [file 12877_2024_5084_MOESM3_ESM.pdf]

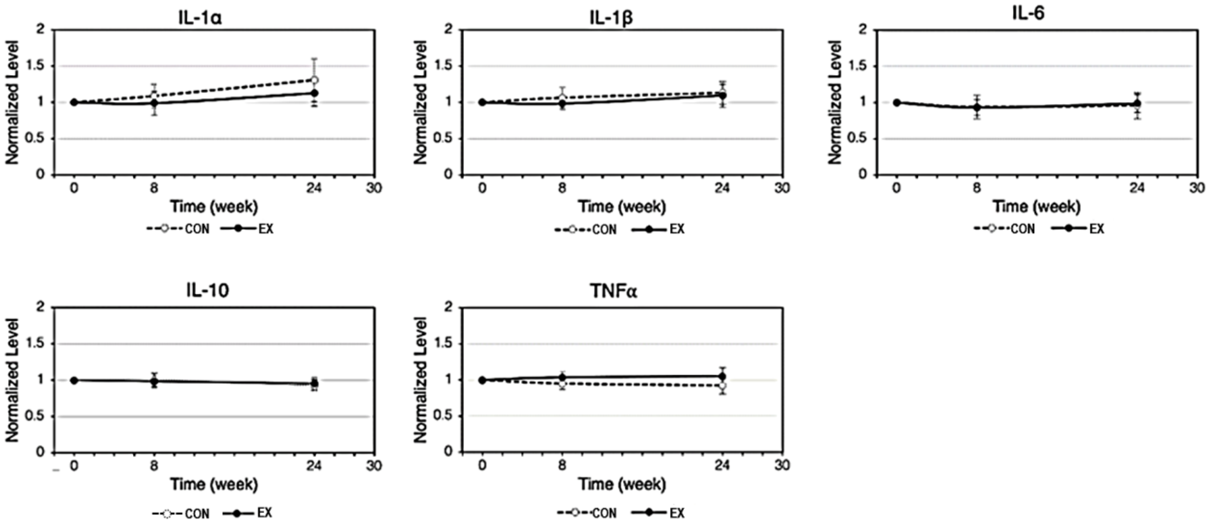

Supplemental Fig. 2. No significant difference was observed for the inflammatory-related cytokines activities for both groups.
