# Supplementary material for: Sustainability of exercise-induced benefits on circulating MicroRNAs and physical fitness in community-dwelling older adults: a randomized controlled trial with follow up
Source: BMC Geriatr. 2024 May 30;24:473. doi: 10.1186/s12877-024-05084-0 (PMC11137894; doi:10.1186/s12877-024-05084-0)
Supplement: Supplementary file 4 — Supplementary Material 4. [file 12877_2024_5084_MOESM4_ESM.pdf]

Supp. Table 1: Effects of supervised exercise training on health-related physical fitness in older community-dwellers.

		EX (n=28)	CON (n=30)	*p* value
**Physical Fitness**				
METs	Baseline	6.84 (6.63-7.05)[‡]	7.00 (6.44-7.55)	0.305
	Week 8	7.07 (6.84-7.30)	7.07 (6.48-7.65)	0.999
	Week 24	7.19 (6.96-7.41)[‡]	7.02 (6.41-7.62)	0.289
Calf circumference, cm	Baseline	33.8 (32.3-35.4)	34.7 (31.0-38.4)[‡]	0.416
	Week 8	33.8 (32.5-35.1)	34.0 (29.3-38.6)[§]	0.907
	Week 24	34.3 (32.9-35.7)	32.3 (28.4-36.2)[‡,§]	0.051
Handgrip strength, kg	Baseline	28.7 (24.2-33.1)	29.2 (19.3-39.1)[†,‡,]	0.854
	Week 8	28.8 (24.7-33.0)	26.7 (17.6-35.8)[†,§]	0.432
	Week 24	30.1 (26.1-34.1)	24.7 (16.6-32.7)[‡,§]	0.033*
5-time sit-to-stand, sec	Baseline	10.8 (9.64-11.9)	11.7 (8.96-14.4)	0.224
	Week 8	10.3 (9.35-11.2)	11.9 (9.19-14.7)	0.021*
	Week 24	10.2 (9.14-11.3)	12.2 (9.31-15.1)	0.011*
Body Composition				
nBFM, %	Baseline	33.3 (30.3-36.4)[†,‡]	31.7 (24.4-39.1)	0.442
	Week 8	31.3 (28.5-34.0)[†,§]	31.6 (25.1-38.1)	0.873
	Week 24	28.4 (25.9-30.9)[‡,§]	33.0 (27.4-38.5)	0.008*
nSKM, %	Baseline	52.9 (52.2-53.6)[†,‡]	54.2 (52.9-55.4)	0.051
	Week 8	55.2 (54.3-56.2)[†,§]	54.3 (53.1-55.5)	0.068
	Week 24	57.5 (56.6-58.4)[‡,§]	54.2 (53.0-55.4)	<0.001*
**MicroRNA**				
miR-21	Baseline	1.00 (1.00-1.01)	1.00 (1.00-1.01)	0.850
	Week 8	1.20 (0.98-1.42)	0.83 (0.25-1.40)	0.030*
	Week 24	2.10 (1.07-3.13)	0.95 (0.13-1.76)	0.044*
miR-126	Baseline	1.01 (1.00-1.01)[†,‡]	1.01 (1.00-1.01)	0.702
	Week 8	1.40 (1.13-1.68)[†]	0.94 (0.15-1.73)	0.039*
	Week 24	3.25 (1.78-4.72)[‡]	1.16 (0.07-2.25)	0.012*
miR-146a	Baseline	1.01 (1.00-1.02)	1.01 (1.00-1.01)	0.512
	Week 8	1.68 (1.15-2.21)	0.88 (0.19-1.57)	0.012*
	Week 24	3.02 (1.51-4.53)	0.87 (0.02-1.73)	0.010*
miR-222	Baseline	1.02 (1.00-1.04)[†,]	1.01 (1.00-1.01)	0.276
	Week 8	1.36 (1.11-1.61)[†]	0.98 (0.49-1.47)	0.025*
	Week 24	2.57 (1.36-3.78)	1.05 (0.27-1.83)	0.029*

Data are presented as mean (95% confidence interval).

Abbreviations: EX: exercise group; CON: control group; MET: metabolic equivalent in 3.5 ml O_2/min/kg; nBFM: normalized body fat mass=(BFM/body weight) ×100%; nSKM: normalized skeletal muscle mass=(SKM/fat free mass)×100%; SET:supervised exercise training.

Main effect for group mixed ANOVA: [*]; main effect for time mixed ANOVA: [†], post-hoc Bonferroni test between baseline and week 8; [‡], post-hoc Bonferroni test between baseline and week 24; [§], post-hoc Bonferroni test between week 8 and 24.
